# [Ru(bpy)_2_(NO)SO_3_](PF_6_), a Nitric Oxide Donating Ruthenium Complex, Reduces Gout Arthritis in Mice

**DOI:** 10.3389/fphar.2019.00229

**Published:** 2019-03-12

**Authors:** Ana C. Rossaneis, Daniela T. Longhi-Balbinot, Mariana M. Bertozzi, Victor Fattori, Carina Z. Segato-Vendrameto, Stephanie Badaro-Garcia, Tiago H. Zaninelli, Larissa Staurengo-Ferrari, Sergio M. Borghi, Thacyana T. Carvalho, Allan J. C. Bussmann, Florêncio S. Gouveia, Luiz G. F. Lopes, Rubia Casagrande, Waldiceu A. Verri

**Affiliations:** ^1^Laboratory of Pain, Inflammation, Neuropathy, and Cancer, Department of Pathology, Londrina State University, Londrina, Brazil; ^2^Department of Organic and Inorganic Chemistry, Federal University of Ceará, Fortaleza, Brazil; ^3^Department of Pharmaceutical Sciences, University Hospital (Health Science Centre), Londrina State University, Londrina, Brazil

**Keywords:** nitric oxide donor, ruthenium, joint pain, oxidative stress, cytokines

## Abstract

Monosodium urate crystals (MSU) deposition induces articular inflammation known as gout. This disease is characterized by intense articular inflammation and pain by mechanisms involving the activation of the transcription factor NFκB and inflammasome resulting in the production of cytokines and oxidative stress. Despite evidence that MSU induces iNOS expression, there is no evidence on the effect of nitric oxide (NO) donors in gout. Thus, the present study evaluated the effect of the ruthenium complex donor of NO {[Ru(bpy)_2_(NO)SO_3_](PF_6_)} (complex I) in gout arthritis. Complex I inhibited in a dose-dependent manner MSU-induced hypersensitivity to mechanical stimulation, edema and leukocyte recruitment. These effects were corroborated by a decrease of histological inflammation score and recruitment of Lysm-eGFP^+^ cells. Mechanistically, complex I inhibited MSU-induced mechanical hypersensitivity and joint edema by triggering the cGMP/PKG/ATP-sensitive K (+) channels signaling pathway. Complex I inhibited MSU-induced oxidative stress and pro-inflammatory cytokine production in the knee joint. These data were supported by the observation that complex I inhibited MSU-induced NFκB activation, and IL-1β expression and production. Complex I also inhibited MSU-induced activation of pro-IL-1β processing. Concluding, the present data, to our knowledge, is the first evidence that a NO donating ruthenium complex inhibits MSU-induced articular inflammation and pain. Further, complex I targets the main physiopathological mechanisms of gout arthritis. Therefore, it is envisaged that complex I and other NO donors have therapeutic potential that deserves further investigation.

## Introduction

Gouty arthritis or gout is the main cause of joint inflammation in men (VanItallie, 2010). Its prevalence has increased in recent years, especially in developed countries in Europe, United States, and Canada, reaching at least 1% of the adult population (Kuo et al., 2015). Patients are affected by agonizing pain and swelling in the joint, which cause disability and considerably impair the quality of life (Roddy et al., 2007; Singh and Strand, 2007). The etiology of gout is related to the increase of serum uric acid levels due to changes in its metabolism, such as exacerbated production or decreased excretion, which means that its solubility is exceeded (Chilappa et al., 2010; Busso and Ea, 2011). The increased concentration of circulating uric acid culminates in deposition of monosodium urate crystals (MSU) in the joints and in periarticular tissues. The deposition of these crystals activate components of the innate immune system and produces intense local inflammatory response (Dieppe et al., 1982; Martinon, 2010) responsible for the symptoms previously mentioned.

The inflammatory response triggered by uric acid crystals involves mechanisms such as increased local oxidative stress by the activation of NADPH oxidases (Busso and So, 2010); leukocyte infiltration, especially neutrophils (Popa-Nita and Naccache, 2010); production of inflammatory mediators, such as tumor necrosis factor alpha (TNF-α) and interleukin (IL) -6 (di Giovine et al., 1991; Choe et al., 2011). In especially, NALP3 inflammasome matures (Martinon et al., 2006) pro-IL-1β into the active form IL-1β, which plays a key role in the development of gouty arthritis (Martinon et al., 2006; So et al., 2007; Dumusc and So, 2015). This pathway is one of the most characteristic innate immune mechanisms of gout.

The pharmacological treatment recommended for acute attacks of gout aims to relieve deleterious symptoms and consists of non-steroidal anti-inflammatory drugs (NSAIDs), colchicine or corticosteroids (Baker and Ralph Schumacher, 2010). In situations of recurrence and chronicity, drug therapy aims to decrease uric acid levels using xanthine-oxidase inhibitors (especially allopurinol) and uricosuric agents (Baker and Ralph Schumacher, 2010). Despite the effectiveness, its use may be accompanied by various side effects such as hypersensitivity reactions, renal and gastrointestinal disorders (Seth et al., 2014; van Echteld et al., 2014; Campochiaro, 2016). Recently, the use of IL-1 inhibitors has proved to be an important pharmacological approach in gout (So et al., 2007) despite expensive costs. In this sense, novel therapeutic approaches have been studied in order to reduce symptoms, control disease and promote improvement of life quality for patients with a reasonable cost.

Nitric oxide (NO) is a key molecule in pain modulation and its therapeutic effect in painful and inflammatory conditions has been demonstrated in different studies through the use of NO donors (Staurengo-Ferrari et al., 2013, 2014a). The ruthenium NO donors inhibit inflammatory pain behaviors induced by formalin and carrageenan by reducing the recruitment of neutrophils (Staurengo-Ferrari et al., 2013), cytokine production and oxidative stress in mice (Staurengo-Ferrari et al., 2014a), and activating the cGMP/PKG/ATP-sensitive potassium channel signaling pathway (Staurengo-Ferrari et al., 2013, 2014a). The nitric oxide donor *cis*-[Ru(bpy)_2_(SO_3_)NO](PF_6_) has protective effect of the gastric mucosa against the damage caused by naproxen or ethanol in mice (Santana et al., 2015).

Considering the evidences of the anti-inflammatory and analgesic effect of NO donors in different animal models, it is plausible the hypothesis that this class of molecules would be effective in the treatment of pain and inflammation in gout. On the other hand, there is also evidence that inhibition of inducible nitric oxide synthase (iNOS) diminishes gout-induced mRNA expression of inflammatory markers and inflammation (Ju et al., 2011). Further, to our knowledge, there is no study evaluating the effect of NO donors in MSU-induced gout inflammation and pain. Therefore, we reason that evaluating the effect of a NO donor in the context of gout is necessary. In this context, this study evaluated the effect and mechanisms of ruthenium NO donor [Ru(bpy)_2_(NO)SO_3_](PF_6_) in the treatment of gout arthritis.

## Materials and Methods

### Animals

Adult male Swiss mice (25–30 g) and LysM-eGFP^+^ C57/BL6 mice (20–25 g) (mice expressing green fluorescent protein under the LysM promoter) obtained from the Universidade Estadual de Londrina (Parana, Brazil) and Ribeirão Preto Medical School (São Paulo, Brazil), respectively, were used to perform the experiments. Mice were housed at 22 ± 2°C in standard polypropylene boxes 41 cm × 34 cm × 16 cm in maximum number of 6 animals randomly per cage, under a 12-h light/12-h dark cycle with access to food and water *ad libitum* and were acclimatized to the laboratory for at least 1 h before testing. The experiments were always conducted at the same time of day and the animals were monitored daily for evaluation of general health status. All experiments were performed with the approval of the Ethics Committee for Animal Research of the Universidade Estadual de Londrina (UEL) (process number 14600.2013.73) and were carried out in accordance with the current guidelines for the care of laboratory animals and the ethical guidelines for investigations of experimental pain in conscious animals (Zimmermann, 1983) and International Association for the Study of Pain (IASP).

### Drugs

The following drugs were obtained from the sources: Glibenclamide (GLB) and KT5823 were obtained from Sigma Aldrich (St. Louis, MO, United States). 1H-[1,2,4]oxadiazolo[4,3,-a]quinoxalin-1-one (ODQ) was purchased from Calbiochem (San Diego, CA, United States). Ruthenium NO donor [Ru(bpy)_2_(NO)SO_3_](PF_6_) was synthesized and used as previously described (Silva et al., 2006). ODQ and KT5823 were diluted in dimethylsulfoxide (DMSO) 2% in saline, and GLB was diluted in Tween 80 5% in saline.

### Ruthenium NO Donor [Ru(bpy)_2_(NO)SO_3_](PF_6_) (Complex I) Synthesis

The ruthenium NO donor [Ru(bpy)_2_(NO)SO_3_](PF_6_) (complex I) synthesis and characterization were performed as previously described (Silva et al., 2006). Aiming to quantify ruthenium in this complex, a methodology proposed by Rowston and Ottaway (1970) was used with modification. For this, a Ru cathode lamp, a flame air-acetylene and flame atomic absorption spectrophotometer Varian, model AA240FS were used. Electrochemical measurements were obtained using a potentiostat/galvanostat manufactured by Bioanalytical Systems, Epsilon model, with a cell containing three electrodes connected as vitreous carbon (working electrode), Ag/AgCl (reference electrode) and platinum (auxiliary electrode). For measurements in acetonitrile, tetrabutylammonium perchlorate (0.1 mol L^-1^) was used as carrier electrolyte and ferrocene was used as internal standard. For measurements in aqueous medium, sodium trifluoroacetate (0.1 mol^-1^, pH 4.7) was used as carrier electrolyte. A similar complex to complex I was synthesized without NO and used as a control in the initial behavior and cell count experiments.

### MSU Crystal Preparation

Monosodium urate crystals were prepared according to the method described previously (Nishimura et al., 1997). Briefly, 800 mg of monosodium urate was dissolved in 155 ml boiling water containing 5 ml 1N NaOH, the pH was adjusted to 7.2 and the solution was cooled gradually by stirring at room temperature. The crystals were collected by centrifugation at 3,000 *g* for 2 min at 4°C. The crystals were evaporated and sterilized by heating at 180°C for 2 h and stored in a sterile environment until use.

### Model of Gout

Joint inflammation characteristic of gout was induced by intra-articular (i.a.) administration of MSU crystals (100 μg/10 μl saline) into the right articular knee joint of mice that were mildly anesthetized. Control animals received an intra-articular injection of 10 μl sterile saline. The dose of MSU was determined in previous study (Ruiz-Miyazawa et al., 2017).

### Electronic Pressure Meter Test of Mechanical Hypersensitivity

Hypersensitivity to mechanical stimulation was tested in mice using an electronic version of the von Frey filaments, as previously reported (Guerrero et al., 2006). Briefly, the test consists of evoking a tibio-tarsal flexion reflex with a hand-held force transducer (electronic von Frey analgesimeter; Insight, Ribeirão Preto, SP, Brazil) adapted with a non-nociceptive tip probe with area size of 4.15 mm^2^. The investigator was trained to apply the tip perpendicularly to the central area of the plantar surface, inducing the flexion of the hind limb joints. The results were expressed as the flexion-elicited withdrawal threshold (in grams). The hypersensitivity to mechanical stimuli was quantified as the change in pressure applied by subtracting the mean of the 3 values obtained in different times after the MSU injection from the mean of the three values observed before MSU injection.

### Edema Assessment

The articular volume was measured with a gauge (Mitutoyo, Suzano, SP, Brazil) before (baseline) and after the intra-articular stimulus with MSU. The edema value was expressed as edema/mm. The values were obtained by subtracting the baseline values from the measurements obtained at each time point (1, 3, 5, 7, and 15 h after i.a. MSU injection).

### Leukocyte Recruitment

After a surgical incision, the knee joint was exposed and washed three times with 5 μL of phosphate-buffered saline (PBS). Total leukocyte counts were determined in a Neubauer chamber after dilution in Turk solution. To distinguish polymorphonuclear (PMNs) from mononuclear cells, differential cell counts were performed using the Fast Panoptic Kit for histological analysis (Laborclin, Pinhais, PR, Brazil) under a light microscope (Olympus Optical Co., Hamburg, Germany). The results are expressed as number of cells × 10^4^ per joint.

### Immunofluorescence Assay

Articular fluid of LysM-eGFP^+^ mice was collected in sterile slides 15 h after MSU i.a. injection into the knee joints using the same procedures presented in sub-section “Leukocyte Recruitment,” and processed for immunofluorescence assay. DAPI fluorescent stain was added to slides for localization of nucleus in each sample. The representative images and quantitative analysis were performed using a confocal microscope (TCS SP8, Leica Microsystems, Mannheim, Germany) (Zarpelon et al., 2016). The intensity of fluorescence was quantified in randomly selected fields of different groups by a blind evaluator. Result is presented as the eGFP fluorescent intensity.

### Histological Analysis

The knee joint of mice was removed 15 h after MSU stimulus after euthanasia. The samples were fixed with 10% paraformaldehyde in PBS, decalcified for at least 20 days with nitric acid 5% and embedded in paraffin for histological analysis. The paraffin sections were stained with hematoxylin and eosin for blinded morphological analysis and scored by a pathologist using light microscopy. The degrees of the following evaluated parameters were: (a) inflammatory infiltrate (from 0 = no inflammation, 1 = mild, 2 = moderate and 3 = severe inflammation); (b) cartilage injury (from 0 = no injury, 1 = mild, 2 = moderate and 3 = severe injury) and (c) vascular proliferation (from 0 = no vascular proliferation, 1 = mild, 2 = moderate and 3 = severe vascular proliferation). The final score was determined by summing the three parameters for each sample expressed as the mean of 5 samples accordingly to the group, and the maximum total score considered was 9. The results are expressed as the median value (variation) for each group of five animals. Images were obtained at 10 and 40× magnification.

### FRAP and ABTS Assays

The tissue antioxidant capacity was determined by their free radical scavenging (ABTS [2,2′-Azinobis-3-ethylbenzothiazoline 6-sulfonic acid] assay) and ferric reducing (FRAP assay) properties. These tests were adapted to a 96-well microplate format as previously described (Campanini et al., 2013). Articular tissue samples were collected 15 h after MSU i.a injection (100 μg/10 μl) and homogenized immediately in ice-cold KCl buffer (500 μl, 1.15% w/v). The homogenates were centrifuged (200 g × 10 min × 4°C), and the supernatants were used in both assays. Diluted ABTS solution (200 μl) was added to 10 μl of sample in each well and the absorbance was measured at 730 nm after 6 min of incubation at 25°C. For FRAP assay, the supernatants (10 μl) were mixed with the freshly prepared FRAP reagent (150 μl) and after incubation at 37°C for 30 min, the absorbance was measured at 595 nm (Multiskan GO Thermo Scientific). The results of FRAP and ABTS assays were equated against a standard Trolox curve (0.02–20 nmol) and expressed as nmol Trolox eq. per mg of tissue.

### GSH Levels Measurement

Articular tissue samples were collected 15 h after MSU i.a injection (100 μg/10 μl/joint) and maintained at -80°C for at least 48 h. The samples were homogenized with 200 μl of 0.02 M EDTA, 25 μl of trichloroacetic acid 50% was added to the homogenate and homogenized three times over 15 min. The mixture was centrifuged (15 min × 1,500 *g* × 4°C) and the supernatant was added to 200 μl of 0.2 M TRIS buffer, pH 8.2, and 10 μl of 0.01M DTNB in 96-well microplate. After 5 min, the absorbance was measured at 412 nm (Multiskan GO, Thermo Scientific). The results are obtained were compared to a standard curve of GSH (Borghi et al., 2014) and expressed as GSH per mg of protein.

### Cytokines Measurement

The knee joint samples were collected 15 h after MSU i.a. injection (100 μg/10 μl/joint) and homogenized in 500 μl of buffer with protease inhibitors followed by centrifugation. The levels of IL-1β, TNF-α, IL-6, and IL-10 were determined by enzyme-linked immunosorbent assay (ELISA) using commercial kits (eBioscience, San Diego, CA, United States). Absorbance was measured at 450 nm (Multiskan GO, Thermo Scientific) and the results are expressed as picograms of cytokines per mg of tissue.

### NF-κB Activation

The knee joint samples were collected 15 h after MSU i.a. injection (100 μg/10 μl/joint) and homogenized in 400 μl of lysis buffer with (Cell Signaling, Danvers, MA, United States) followed by centrifugation. The determination of phosphorylated NF-κB p65 subunit (activated) and total levels of NF-κB p65 subunit were performed using ELISA PathScan Kits (Cell Signaling, Danvers, MA, United States) according to the manufacturer’s directions. Absorbance was measured at 450 nm (Multiskan GO, Thermo Scientific) and the results are expressed as IOD ratio (total NF-κB p65/p-NF-κB p65).

### Western Blot Analysis

The knee joint samples were collected 15 h after MSU i.a. injection (100 μg/10 μl/joint) and homogenized in 400 μl and homogenized in RIPA buffer (Sigma-Aldrich, St. Louis, MO, United States) containing protease and phosphatase inhibitors (Cell Signaling Technology, Beverly, MA, United States). The lysates were then homogenized and centrifuged (0.5 g for 10 min at 4°C). The protein concentrations of the lysate were determined using a BCA Protein Assay kit (Pierce, Rockford, IL, United States), and 100 μg of protein was loaded for each lane. The protein extracts were separated by SDS-PAGE on 15% gel and transferred to a nitrocellulose membrane (GE Healthcare-Amersham, Pittsburgh, PA, United States). Membranes were then incubated in blocking buffer [5% bovine serum albumin (BSA) or 5% non-fat milk in Tris-buffered saline (TBS) with Tween 20] for 2 h at room temperature and incubated overnight at 4°C in the presence of primary antibody diluted in 5% BSA in TBS with Tween 20 or 5% non-fat milk. The primary antibodies used to Western blot were IL-1β (1:500; Cell Signaling Technology, Beverly, MA, United States) and β-Actin (1:5000; Santa Cruz Biotechnology). After washing in TBS with Tween 20, the membrane was incubated with a HRP-conjugated secondary antibody (1:5000; Jackson Immuno Research, West Grove, PA, United States) for 2 h at room temperature. Protein was visualized by chemiluminescence with Luminata^TM^ Forte Western HRP Substrate (Merck Millipore Corporation, Darmstadt, Alemanha). The molecular protein mass was confirmed by PageRuler^TM^ Prestained Protein Ladder (Thermo Scientific, Rockford, IL, United States). The membranes were reprobed with antibody against β-Actin to be used as loading control. Densitometric data were measured using Scientific Imaging Systems (Image Lab 3.0 software; Bio-Rad Laboratories, Hercules, CA, United States).

### Quantitative Polymerase Chain Reaction (qPCR)

Mice were euthanized 15 h after the i.a. injection of MSU, the knee joint samples were collected, homogenized in 500 μl of Trizol reagent and centrifuged (12,000 rcf, 15 min, 4°C). Total RNA was extracted using the SV Total RNA Isolation System (Promega) (Verri et al., 2008) and measured with a spectrophotometer and the wavelength absorption ratio (260/280 nm) was between 1.8 and 2.0 for all preparations. qPCR was performed in a LightCycler Nano Instrument (Roche, Mississauga, ON, United States) sequence detection system using the Platinum SYBR Green qPCR SuperMix UDG (Invitrogen, United States). The mRNA level of glyceraldehyde 3-phosphate dehydrogenase (GAPDH) was used as an internal control. The primers used were Gapdh forward: CAT ACC AGG AAA TGA GCT TG, reverse: ATG ACA TCA AGA AGG TGG TG; gp91phox (NADPH oxidase sub-unity), forward: AGC TAT GAG GTG GTG ATG TTA GTG G, reverse: CAC AAT ATT TGT ACC AGA CAG ACT TGA G; nlrp3, forward: AGC TAT GAG GTG GTG ATG TTA GTG G, reverse: CAC AAT ATT TGT ACC AGA CAG ACT TGA G; pro-il-1β, forward: GAA ATG CCA CCT TTT GAC AGT G, reverse: TGG ATG CTC TCA TCA GGA CAG; cyclooxygenase-2 (COX-2), forward: GTG GAA AAA CCT CGT CCA GA, reverse: GCT CGG CTT CCA GTA TTG AG. The SYBR green PCR Master Mix was used according to the manufacturer’s instructions.

### Bone Marrow-Derived Macrophages (BMDMs) Culture and Inflammasome Activation Assay

Bone marrow cells were obtained from the aspiration of femora and tibiae of mice C57BL/6 mice (8 weeks old) and cultured in RPMI 1640 medium containing 10% FBS and 15% L929 cell conditioned medium. BMDM were harvested at day 7 and plated at the density of 1.5 × 10^5^ cells/well in 96-well plate. BMDM were stimulated with 500 ng/mL *Escherichia coli* LPS (Santa Cruz Biotechnology) and 3 h later treated with complex I (0.1, 1, 10, or 100 μM) 30 min before MSU stimulation (450 μg/ml) of NLRP3 inflammasome activation as described previously (Martinon et al., 2006). Supernatants were collected 5 h after MSU stimulation and IL-1β concentration quantitated by ELISA.

### Neuronal Cultures and Calcium Imaging

Mice received MSU or saline i.a. injection (100 μg/10 μl/joint) and 15 h after, L4-L6 dorsal root ganglia (DRG) were dissected into DMEM (Sigma) and digested in collagenase/dispase solution (5 mM CaCl_2_) at 37°C for 75 min. DRG cells were triturated with pipette tips, centrifuged and resuspend in DMEM + 10% FBS, and incubate on laminin-coated cell culture dishes for 60–75 min at 37°C. The laminin was removed, DMEM medium was added to the plate and cells were incubated overnight at 37°C. For calcium imaging, cells were loaded with 2 μM of Fluo-4 in DMEN, incubated for 45 min 37°C, washout with HBSS and imaged in Confocal Microscope (TCS SP8, Leica Microsystems, Mannheim, Germany). After the 2 min of initial reading, the cells were treated with complex I (100 μM) or vehicle and observed for 5 min, capsaicin (1 μM) was added, and 2 min after, 40 mM KCl was added, as previously described (Chiu et al., 2013) and the calcium flux was analyzed by the mean fluorescence measured with the LAS X Software (Leica Microsystems). The number of cells responsive to capsaicin relative to the total was analyzed in saline, MSU and vehicle and MSU and complex I groups.

### Experimental Protocols

Mice were randomly divided into groups with *n* = 6 (or 5 in the histopathology studies) and treated with complex I (0.3, 1, or 3 mg/kg/saline, s.c.), empty NO complex I (1 mg/kg/saline, s.c) or vehicle 30 min before MSU (100 μg/10 μl/joint). The mechanical hypersensitivity and edema were measured 1, 3, 5, 7, and 15 h after the stimulus injection. Sample collection was always at the 15th hour after mice were terminally anesthetized on an isoflurane chamber. Mechanical hypersensitivity and edema were evaluated 1, 3, 5, 7, and 15 h after the MSU stimulus. Doses, routes of administration and time of treatment were based on previous studies (Staurengo-Ferrari et al., 2013; Ruiz-Miyazawa et al., 2017). No animal showed signs of weakness or adverse effects from stimuli or treatments, and no animal was required to be excluded during project development. All experiments were blinded and performed twice, which is essential to demonstrate the reproducibility and replicability of the data.

### Statistical Analysis

The presented results are representative of two independent experiments and are expressed as the mean ± SEM (*n* = 6 per group per experiment). Two-way ANOVA with repeated measures was used to compare the effect of treatments at different time points. The factors analyzed were treatments, time and treatment versus time interaction. One-way ANOVA followed by Bonferroni’s test was performed to evaluate the differences between responses at each time-point. One-way ANOVA followed by Newman–Keuls test was performed to evaluate the histopathological score. Statistical differences were considered to be significant at *P* < 0.05. The analyzes were performed by GraphPad Prism 7.00 software.

## Results

### Ruthenium NO Donor [Ru(bpy)_2_(NO)SO_3_](PF_6_) (Complex I) Reduces MSU-Induced Mechanical Hypersensitivity and Joint Edema

**FIGURE 1:**
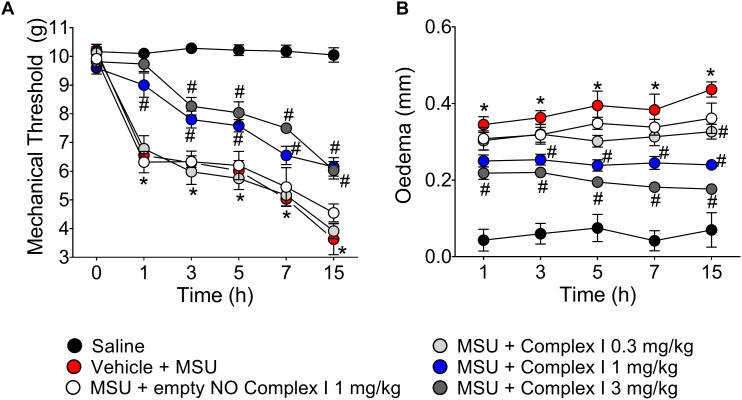
Ruthenium NO donor [Ru(bpy)_2_(NO)SO_3_](PF_6_) (complex I) inhibits MSU-induced mechanical hypersensitivity and joint edema. Mice were treated with complex I (0.3, 1, or 3 mg/kg/saline, s.c.) or empty NO complex I (1 mg/kg/saline, s.c.) or vehicle 30 min prior to intra-articular stimulation with MSU (100 μg/10 μl). Mechanical hypersensitivity **(A)** and Edema **(B)** were evaluated at the intervals of 1, 3, 5, 7, and 15 h after the stimulus with MSU. Results are expressed as mean ± SEM (*n* = 6 per group per experiment, representative of two experiments). ^∗^*p* < 0.05 compared to saline group and ^#^*p* < 0.05 compared to MSU + vehicle. Two-way ANOVA followed by Tukey’s test.

The first set of experiments present at Figure 1 started by addressing whether complex I would present a dose-dependent effect over disease parameters, which were knee joint pain (e.g., mechanical hypersensitivity) and edema. A dose-response curve is important to select a dose to verify the mechanisms of the drug under test at a pharmacologically active dose. To this end, 30 animals were randomly distributed in 5 groups of 6 animals and the experiment was performed twice, totaling 60 animals. NO induces analgesia or nociception depending on the dose (Cury et al., 2011). The following experiments were designed to determine if the ruthenium NO donor [Ru(bpy)_2_(NO)SO_3_](PF_6_) (complex I) can induce analgesia and reduce inflammation. Mice were treated with complex I (0.3, 1, or 3 mg/kg/saline, s.c.), empty NO complex I (1 mg/kg/saline, s.c.) or vehicle 30 min before MSU (100 μg/10 μl/joint) or saline and hypersensitivity to mechanical stimulation (Figure 1A) and edema (Figure 1B) were evaluated at 1, 3, 5, 7, and 15 h after MSU injection. Intra-articular injection of MSU crystals produced significant reduction of the mechanical withdrawal threshold at all evaluated intervals when compared to saline group. When compared to the MSU control group, the pretreatment with the 0.3 mg/kg dose of complex I was not able to produce significant changes of mechanical withdrawal threshold in any of the evaluated time points. On the other hand, both the 1 and 3 mg/kg doses of complex I significantly decreased the mechanical hypersensitivity at all time points. There was no significant effect difference between the two groups treated with the higher doses. A point to emphasize is that these two doses were able to totally inhibit the mechanical hypersensitivity induced by the MSU in the 1st hour after the stimulus, resembling the mechanical withdrawal threshold to that that observed in the saline group (Figure 1A). As for edema, significant increase in joint volume was confirmed after MSU injection for up to 15 h when compared to saline group. Animals treated with the lowest dose of complex I (0.3 mg/kg) showed significant reduction in edema only at the 5th and 15th hour after the stimulus when compared to the group stimulated with MSU and treated with vehicle. The 1 mg/kg dose significantly inhibited the edema up to the 15 h after the stimulus, except in the 1st hour, whereas the dose of 3 mg/kg presented significant effect on the reduction of edema at all time points (Figure 1B). The treatment with the empty NO complex I (1 mg/kg) had no effect on mechanical hypersensitivity or joint edema at the evaluated intervals.

### Complex I Reduces MSU-Induced Leukocyte Recruitment

**FIGURE 2:**
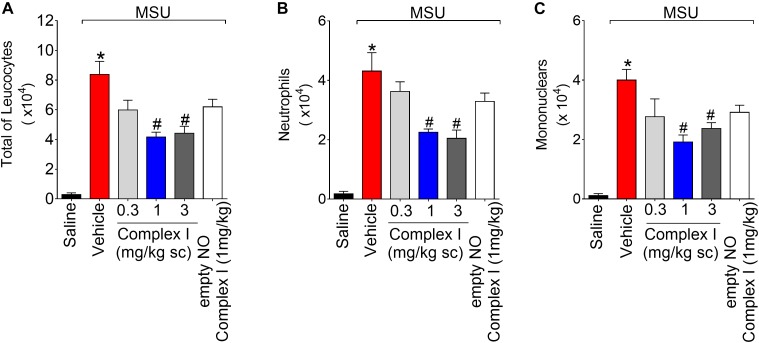
Complex I inhibits MSU-induced leukocyte recruitment in the knee joint cavity. Mice were treated with complex I (0.3, 1, or 3 mg/kg/saline, s.c.) or empty NO complex I (1 mg/kg/saline, s.c.) or vehicle 30 min prior to intra-articular stimulation with MSU (100 μg/10 μl). Total leukocytes **(A)** and differential counts of polymorphonuclear **(B)** and mononuclear **(C)** cells in synovial fluid were performed 15 h after MSU stimulus. Results are expressed as mean ± SEM (*n* = 6 per group per experiment, representative of two experiments). ^∗^*p* < 0.05 compared to saline group and ^#^*p* < 0.05 compared to MSU + vehicle. One-way ANOVA followed by Tukey’s test.

At Figure 2 we show the experiments regarding the recruitment of leukocytes and potential inhibitory dose-dependent effect of complex I. During inflammation, leukocytes are recruited from the vascular circulation toward the primary inflammatory foci, which in the present experimental condition is the knee joint that received MSU crystals administration. At the inflammatory foci, leukocytes phagocyte MSU crystals and amplify the inflammatory response (So and Martinon, 2017). Therefore, limiting the leukocyte recruitment is an important approach to reduce inflammation. For this experiment the same 30 animals described in the previous result (item 3.1) were used. The experiment was performed twice, totaling 60 animals. Mice were treated with complex I (0.3, 1, or 3 mg/kg/saline, s.c.) or vehicle 30 min before MSU (100 μg/10 μl/joint) and knee joint washes were collected 15 h after stimulus injection. MSU induced an increase of total leukocytes (Figure 2A), neutrophils (Figure 2B), and mononuclear cells (Figure 2C) counts compared to saline negative control group. Pretreatment with complex I at the dose of 0.3 mg/kg did not produce a significant change in the number of cells in the joint cavity when compared to the MSU positive control group. In the group treated with the dose of 1 mg/kg of complex I, a significant reduction of total leukocytes (54%) and mononuclear cells (51.5%) was observed, whereas the treatment with 3 mg/kg of complex I significantly reduced the total leukocytes (47%) and neutrophils (45.2%) in the joint cavity when compared to the MSU and vehicle groups. Similarly to Figure 1, the treatment with the empty NO complex I (1 mg/kg) showed no significant effect on the total leukocytes, neutrophils or mononuclear cells recruitment. Based on the results obtained in Figure 1, 2, the dose of complex I of 1 mg/kg was selected for the following experiments and no further experiments were performed with the empty NO complex I to reduce the number of animals since it was shown to be inactive. Furthermore, the results of Figure 1, 2 show that complex I reduced MSU crystals-triggered inflammatory pain hypersensitivity, edema and leukocyte recruitment.

### Complex I Reduces MSU-Induced Recruitment of LysM-eGFP^+^ Cells in the Knee Joint

**FIGURE 3:**
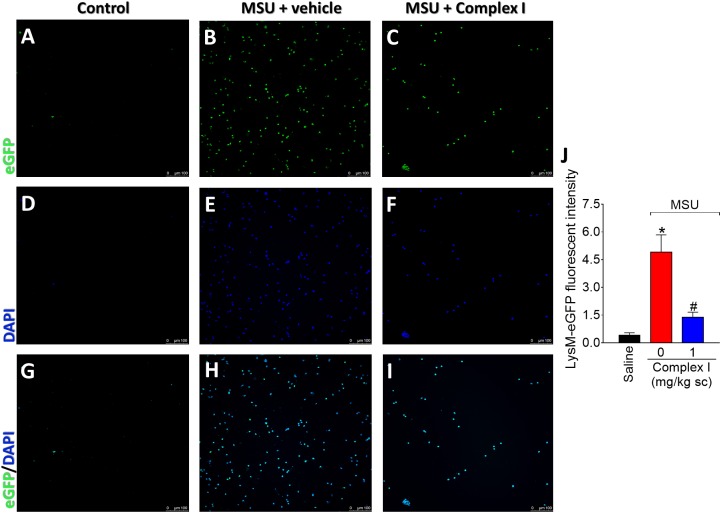
Complex I inhibits MSU-induced neutrophil recruitment to the knee joint. Lysm-eGFP^+^ mice were treated with complex I (1 mg/kg/saline, s.c.) or vehicle 30 min prior to intra-articular stimulation with MSU (100 μg/10 μl) and after 15 h synovial fluid was collected to evaluate the cellular migration (fluorescence intensity). **(A–I)** Show representative images of articular washes of control **(A,D,G)**, MSU + vehicle **(B,E,H)** and MSU + complex I 1 mg/kg **(C,F,I)** and **(J)** shows the mean ± SEM of fluorescence intensity in arbitrary units. Results are expressed as mean ± SEM (*n* = 6 per group per experiment, representative of two experiments). ^∗^*p* < 0.05 compared to saline group and ^#^*p* < 0.05 compared to MSU + vehicle. One-way ANOVA followed by Tukey’s test.

The LysM-eGFP^+^ mice had the enhanced green fluorescent protein gene inserted into the murine lysozyme M (LysM). The LysM enzyme is expressed by macrophages and neutrophils (Faust et al., 2000). Therefore, in the present experimental condition, the LysM-eGFP^+^ mouse gave information on the recruitment of macrophages and neutrophils to the inflamed knee joints. It is important to point out that macrophages and neutrophils are considered essential cells in gout arthritis (So and Martinon, 2017). For this experiment 18 animals were randomly distributed in three groups of six animals. The experiment was performed twice, totaling 36 animals. LysM-eGFP^+^ C57/BL6 mice were treated with complex I (1 mg/kg/saline, s.c.) or vehicle 30 min before MSU (100 μg/10 μl/joint) and the articular fluid was collected 15 h after for the evaluation of the recruitment of LysM-eGFP^+^ cells. MSU induced significant increase of LysM-eGFP^+^ cells in the knee joint washes, which was inhibited by the treatment with complex I (Figure 3). Figure 3A–I show representative images and Figure 3J shows the mean ± SEM of fluorescence intensity in arbitrary units. These results confirm that complex I reduces MSU-induced cell recruitment to the knee joint, specifically, LysM-eGFP^+^ cells.

### Complex I Reduces MSU-Induced Knee Joint Synovitis

**FIGURE 4:**
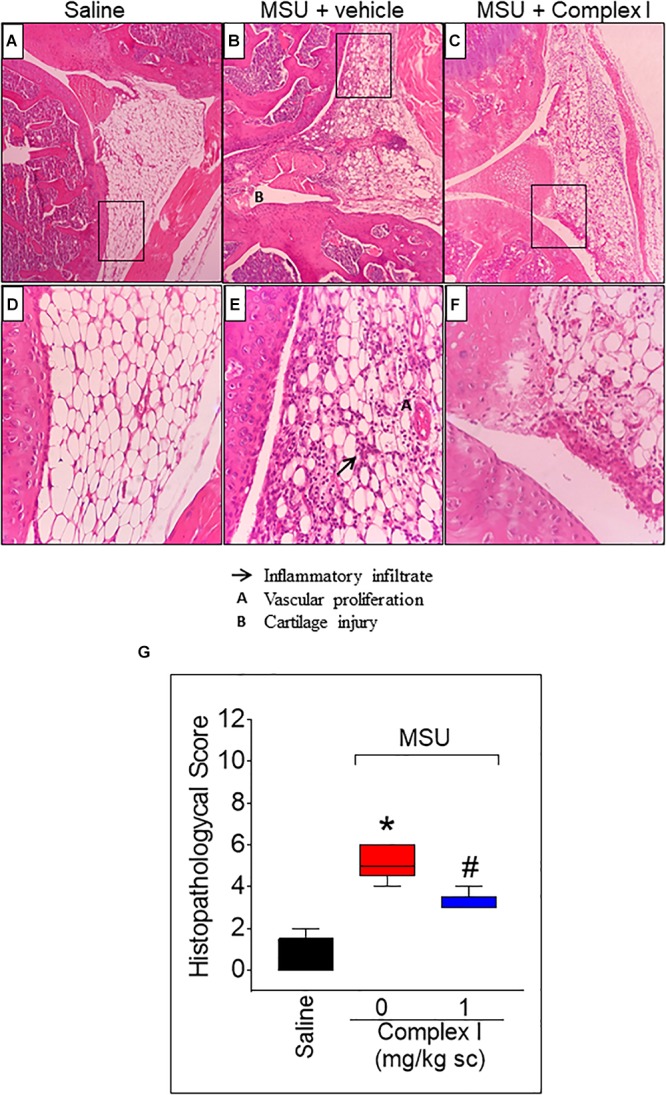
Effect of complex I treatment on histopathology profile alterations in synovial tissue of MSU-induced arthritis in mice. Mice were treated with complex I (1 mg/kg/saline, s.c.) or vehicle 30 min prior to intra-articular stimulation with MSU (100 μg/10 μl) and after 15 h knee joints samples were collected to the histological analysis and score. The sections were stained with Hematoxylin – Eosin. Original magnification 10×**(A–C)** and 40×**(D–F)** and the figures are representative of all experiments. The groups are represented as saline **(A,D)**, MSU + vehicle **(B,E)**, MSU + complex I **(C,F)**. The panel **(G)** indicates the histopathological score. The parameters analyzed were (arrow) inflammatory infiltration; **(A)** vascular proliferation; and **(B)** cartilage injury. Results are expressed as mean ± SEM (*n* = 5 per group per experiment, representative of two experiments). ^∗^*p* < 0.05 compared to saline group and ^#^*p* < 0.05 compared to MSU + vehicle. One-way ANOVA followed by Newman–Keuls test.

Inflammation of the synovial tissue is a disease characteristic of arthritis (Hitchon and El-Gabalawy, 2011). Thus, it is important to evaluate whether a drug candidate to treat arthritis reduces synovitis. For this experiment 15 animals were randomly distributed in 3 groups of 5 animals. The experiment was performed twice, totaling 30 animals Following the same protocol as for Figure 3, knee joint samples were collected 15 h after MSU injection, and sections were stained with Hematoxylin and Eosin. The score was determined by the sum of the 3 parameters: inflammatory infiltrate, cartilage injury and vascular proliferation. The saline group presented normal histological characteristics (Figure 4A,D,G) while the MSU group presented significant increase of inflammatory infiltrate, cartilage injury and vascular proliferation (Figure 4B,E). Treatment with complex I significantly reduces the severity of the histological parameters evaluate (Figure 4C,F).

### Complex I Reduces MSU-Induced Mechanical Hypersensitivity and Joint Edema by Activating the cGMP/PKG/ATP-Sensitive Potassium Channel Pathway

Nitric oxide is known to reduce pain and inflammation by activating the cGMP/PKG/ATP-sensitive potassium channel pathway (Staurengo-Ferrari et al., 2013). Thus, as a NO donor was under test, it was important to determine whether complex I would exert its effects by activating the cGMP/PKG/ATP-sensitive potassium channel pathway. To this end, inhibitors of cGMP (ODQ), PKG (KT5823) and ATP-sensitive potassium channel (GLB) were used (Staurengo-Ferrari et al., 2013). For this experiment 30 animals were randomly distributed in 5 groups of 6 animals and the experiment was performed twice, totaling 60 animals. Mice were treated with ODQ (0.3 mg/kg, i.p.), KT5823 (0.5 μg/animal, i.p.) or GLB (0.3 mg/kg, i.p.) 30, 5, and 45 min, respectively, before the treatment with complex I (1 mg/kg/saline, s.c.) or vehicle. Mechanical hypersensitivity and joint edema were evaluated at 1, 3, 5, 7, and 15 h after MSU stimulus (Supplementary Figure S1). The complex I reduction of MSU-induced mechanical hypersensitivity was prevented by ODQ treatment at 3, 5, and 7 h (Supplementary Figure S1A), by KT5823 (Supplementary Figure S1B) or GLB (Supplementary Figure S1C) treatment at 3 and 5 h. The complex I reduction of MSU-induced knee edema was prevented by ODQ and KT5823 treatments at 5h (Supplementary Figures S1D,E), and GLB treatment at 5 and 7 h (Supplementary Figure S1F). The inhibitors ODQ, KT5823 and GLB did not alter *per se* the mechanical hypersensitivity or edema induced by MSU. Thus, complex I reduced the pain and edema triggered by MSU crystals by activating the cGMP/PKG/ATP-sensitive potassium channel pathway.

### Complex I Inhibits MSU-Induced Oxidative Stress in the Knee Joint

**FIGURE 5:**
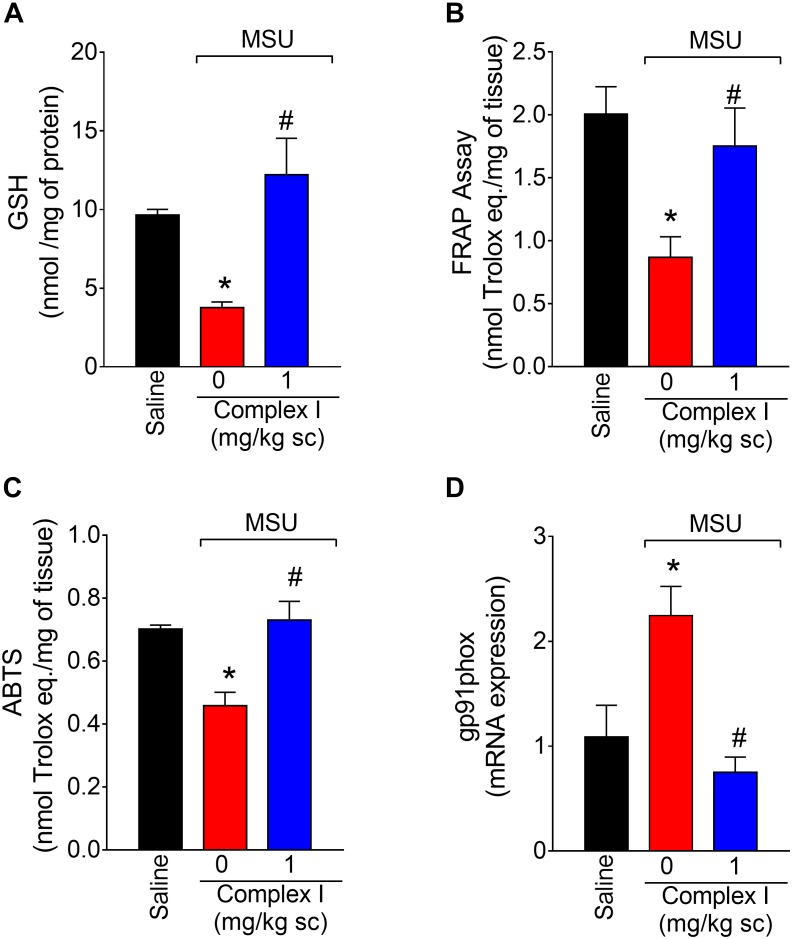
Complex I increases antioxidant defenses in MSU-induced arthritis. Mice were treated with complex I (1 mg/kg/saline, s.c.) or vehicle 30 min prior to intra-articular stimulation with MSU (100 μg/10 μl) and after 15 h knee joints samples were collected to the GSH **(A)**, FRAP **(B)** and ABTS **(C)** assays and gp91phox **(D)** mRNA expression evaluation. Results are expressed as mean ± SEM (*n* = 6 per group per experiment, representative of two experiments). ^∗^*p* < 0.05 compared to saline group and ^#^*p* < 0.05 compared to MSU + vehicle. One-way ANOVA followed by Tukey’s test.

Oxidative stress participates in inflammation by amplifying intracellular signaling pathways involved in promoting inflammation such as the NF-κB. Furthermore, oxidative stress causes tissue damage. In this sense, reducing oxidative stress diminishes disease severity (Del Carlo and Loeser, 2002; Ju et al., 2011; Staurengo-Ferrari et al., 2014a). To verify whether complex I would affect MSU crystals-triggered oxidative stress, 18 animals were randomly distributed in 3 groups of 6 animals. The experiment was performed twice, totaling 36 animals. Mice were treated with complex I (1 mg/kg/saline, s.c.) or vehicle 30 min before MSU (100 μg/10 μl/joint) and the knee joint samples were collected 15 h after for the GSH (reduced glutathione), FRAP (ferric reducing ability potential), ABTS (free-radical scavenging ability) and gp91phox mRNA expression evaluation. MSU significantly deplete the GSH (Figure 5A) and reduced the antioxidant activity in the FRAP (Figure 5B) and ABTS (Figure 5C) assays in the joint when compared to the saline group. Complex I treatment reversed MSU effects restoring the GSH, FRAP, and ABTS to basal levels. The gp91phox mRNA expression was evaluated by qPCR. Complex I significantly inhibited MSU-induced mRNA expression gp91phox (Figure 5D). These data indicate that complex I reduced MSU crystals-triggered oxidative stress.

### Complex I Modulates MSU-Induced Cytokine Production in the Knee Joint

**FIGURE 6:**
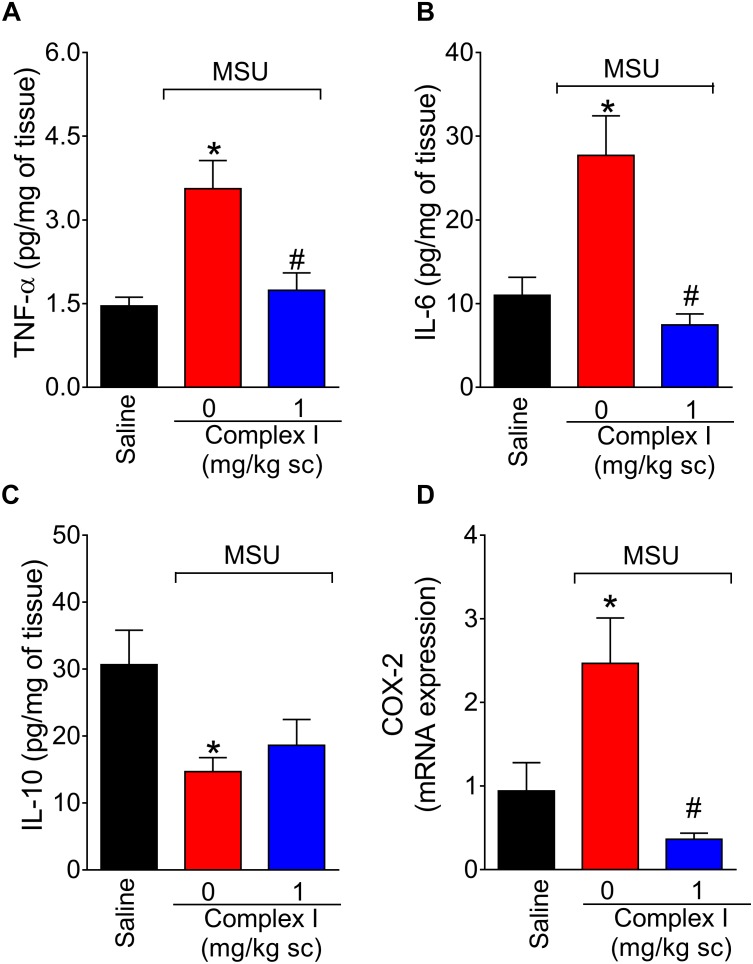
Complex I reduces MSU-induced cytokine production and cox-2 mRNA expression in the knee joint. Mice were treated with complex I (1 mg/kg/saline, s.c.) or vehicle 30 min prior to intra-articular stimulation with MSU (100 μg/10 μl) and after 15 h knee joints samples were collected to measurement of pro-inflammatory TNF-α **(A)** and IL-6 **(B)**, anti-inflammatory IL-10 **(C)** cytokines and cox-2 mRNA expression **(D)**. Results are expressed as mean ± SEM (*n* = 6 per group per experiment, representative of two experiments). ^∗^*p* < 0.05 compared to saline group and ^#^*p* < 0.05 compared to MSU + vehicle. One-way ANOVA followed by Tukey’s test.

Cytokines are cellular products involved in the orchestration of pain and inflammation (Verri et al., 2006). Cytokines such as TNF-α and IL-6 have pro-inflammatory roles. On the other hand, cytokines such as IL-10 have anti-inflammatory roles. Therefore, we tested whether complex I would affect cytokine production. The experiment was performed twice, totaling 36 animals. For each repetition 18 animals were randomly distributed in 3 groups of 6 animals. For the assessment of the levels of cytokines, mice were treated with complex I (1 mg/kg/saline, s.c.) or vehicle 30 min before MSU (100 μg/10 μl/joint) and the knee joint samples were collected 15 h after for the quantification of TNF-α (Figure 6A), IL-6 (Figure 6B), and IL-10 (Figure 6C) by ELISA. The levels of pro-inflammatory cytokines TNF-α and IL-6 were significantly higher in the MSU group, whereas the levels of anti-inflammatory cytokine IL-10 were decreased when compared to saline. Treatment with complex I (1 mg/kg/saline, s.c.) significantly reduced the levels of TNF-α and IL-6 without altering IL-10 level.

### Complex I Inhibits MSU-Induced Cox-2 mRNA Expression

COX-2 is an inducible enzyme involved in the production of prostanoids and also a target of non-steroidal anti-inflammatory drugs (Lee et al., 2005). Therefore, reducing COX-2 expression is an important analgesic and anti-inflammatory mechanism (Lee et al., 2005. Thus, the effect of complex I over cox-2 mRNA expression during gout arthritis was tested. For this experiment 18 animals were randomly distributed in 3 groups of 6 animals. The experiment was performed twice, totaling 36 animals. The cox-2 mRNA expression was evaluated by qPCR 15 h after MSU injection (100 μg/10 μl/joint) in knee joint samples of mice pre-treated with complex I (1 mg/kg/saline, s.c., 30 min) or vehicle. Complex I significantly inhibited MSU-induced cox-2 mRNA expression (Figure 6D).

### Complex I Reduces MSU-Induced NF-κB Activation

**FIGURE 7:**
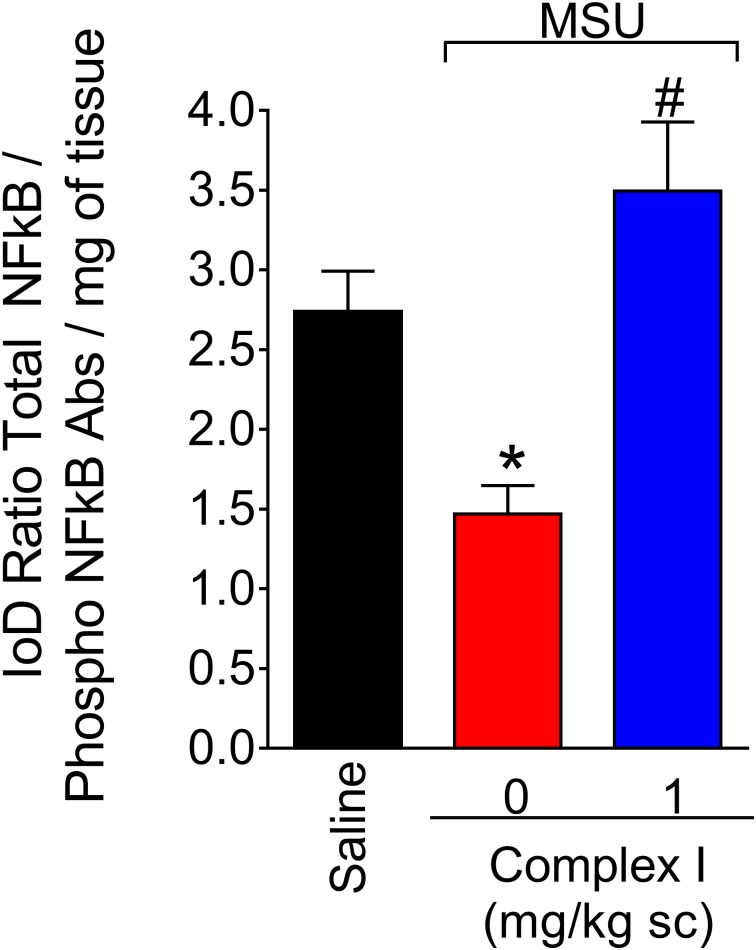
Complex I reduces MSU-induced NF-κB activation in the knee joint. Mice were treated with complex I (1 mg/kg/saline, s.c.) or vehicle 30 min prior to intra-articular injection of MSU (100 μg/10 μl) and after 15 h knee joints samples were collected to measurement of phosphorylated and total NF-κB. Results are expressed as mean ± SEM (*n* = 6 per group per experiment, representative of two experiments). ^∗^*p* < 0.05 compared to saline group and ^#^*p* < 0.05 compared to MSU + vehicle. One-way ANOVA followed by Tukey’s test.

The transcription factor NF-κB regulates the expression of varied inflammatory molecules including COX-2, gp91phox, and cytokines (Pahl, 1999; Anrather et al., 2006). Thus, considering the results of Figure 5, 6, the effect of complex I on NF-κB activation in gout arthritis was tested. For this experiment 18 animals were randomly distributed in 3 groups of 6 animals. The experiment was performed twice, totaling 36 animals. Mice were treated with complex I (1 mg/kg/saline, s.c.) or vehicle 30 min before MSU (100 μg/10 μl/joint) and the knee joint samples were collected 15 h after for evaluation of the levels of total and phosphorylated NF-κB p65 subunit ratio (Figure 7). MSU induced significant activation of NF-κB (a decrease of total and phosphorylated NF-κB p65 subunit ratio) compared to saline control group, and treatment with complex I significantly inhibited MSU-induced NF-κB activation in the knee joint.

### Complex I Inhibits MSU-Induced IL-1β Production and Prevented MSU-Induced Inflammasome Activation

**FIGURE 8:**
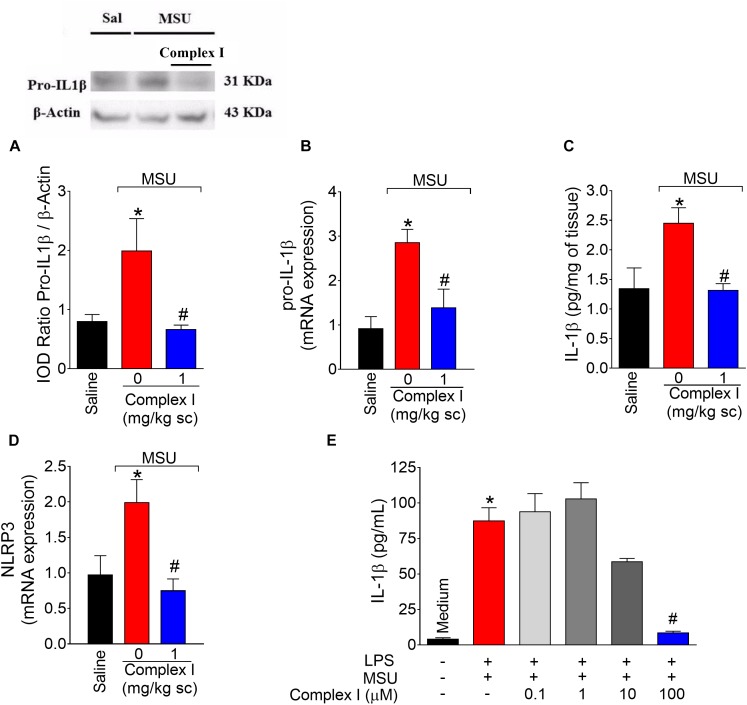
Complex I inhibits MSU-induced IL-1β up-regulation and inflammasome activation. Mice were treated with complex I (1 mg/kg/saline, s.c.) or vehicle 30 min prior to intra-articular injection of MSU (100 μg/10 μl) and after 15 h knee joints samples were collected to measurement of pro-IL-1β (western blot), **(A)**; pro-il-1β mRNA expression (RT-qPCR), **(B)**; IL-1β (ELISA), **(C)** and nlrp3 mRNA expression (RT-qPCR), **(D)**. LPS (500 ng/ml)-primed BMDM were treated with Complex I (0.1, 1, 10, or 100 μM) 30 min before stimulation with MSU (450 μg/ml) during 5 h and the IL-1β concentration (ELISA) in the culture supernatant was measured by ELISA **(E)**. Results are expressed as mean ± SEM (*n* = 6 per group per experiment, representative of two experiments). ^∗^*p* < 0.05 compared to saline group and ^#^*p* < 0.05 compared to MSU group. One-way ANOVA followed by Tukey’s test.

The NLRP3 inflammasome is a molecular platform responsible for the maturation of pro-IL-1β into active IL-1β in gout arthritis (Martinon et al., 2006; Dumusc and So, 2015). Thus, experiments were conducted to determine whether complex I would act by interfering with this important physiopathological mechanism of gout arthritis. We started by western blot analysis that were performed twice, totaling 36 animals. For each repetition 18 animals were randomly distributed in 3 groups of 6 animals. For qPCR experiments, we used the same samples described for cox-2 mRNA expression (Figure 6D). Mice were treated with complex I (1 mg/kg/saline, s.c.) or vehicle 30 min before MSU (100 μg/10 μl/joint) and the knee joint samples were collected 15 h after for evaluation of pro-IL-1β expression by western blot (Figure 8A), pro-il-1β mRNA expression by qPCR (Figure 8B), IL-1β production by ELISA (Figure 8C) and nlrp3 mRNA expression by qPCR (Figure 8D). MSU significantly increased pro-IL-1β expression and IL-1β production in the knee joint, which were inhibited by complex I. Complex I significantly inhibited MSU-induced mRNA expression of the pro-il-1β and nlrp3. Complex I inhibited MSU-induced IL-1β release by macrophages (Figure 8E). BMDM were stimulated with LPS (500 ng/ml) 3 h before treatment with Complex I (0.1, 1, 10, or 100 μM). 30 min after Complex I treatment, BMDM were stimulated with MSU crystals (450 μg/ml). IL-1β concentration in the culture supernatant was measured by ELISA 5 h after MSU stimulus. Complex I at the concentration of 100 μM significantly inhibited the increase of levels of IL-1β induced by LPS (first signal) and MSU (second signal) stimulation in BMDM supernatant, preventing the activation of NLRP3 inflammasome by MSU.

### Complex I Inhibits MSU-Induced TRPV1-Dependent Activation of DRG Nociceptor Neurons

**FIGURE 9:**
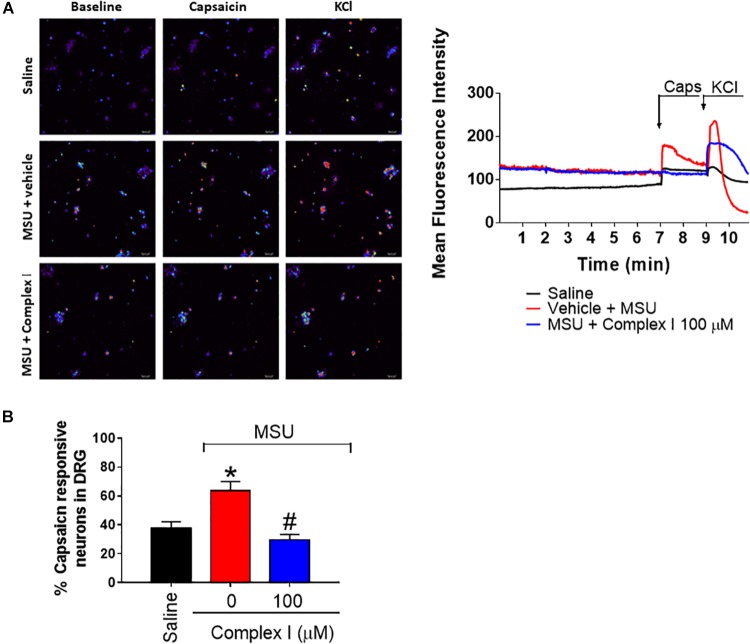
Complex I inhibits MSU-induced TRPV1-dependent DRG nociceptor neurons activation. The *in vivo* injection of MSU (100 μg/10 μl/joint) increases the basal calcium detection in DRG neurons and also the responsiveness to capsaicin (TRPV1 agonist) compared to saline negative control group. Treatment with complex I significantly reduced the intensity of calcium detection **(A)** and the percentage of cells responding to capsaicin **(B)**. Results are expressed as mean ± SEM (*n* = 5 per group per experiment, representative of two experiments). ^∗^*p* < 0.05 compared to saline group and ^#^*p* < 0.05 compared to MSU group. One-way ANOVA followed by Tukey’s test.

During gout arthritis there is an increase of TRPV1-dependent activation of primary nociceptor sensory neurons (Hoffmeister et al., 2011). The cellular bodies of these neurons are in the DRG and their activity can be assessed using the fluorescent probe to detect calcium influx as a parameter of neuronal activation (Yu et al., 2004). For this experiment 18 animals were randomly distributed in 3 groups of 6 animals. The experiment was performed twice, totaling 36 animals. DRG neurons from mice that received MSU or saline i.a. injection (100 μg/10 μl/joint) *in vivo* were cultured. *In vitro* treatment with complex I (100 μM) or vehicle was performed 5 min prior to stimulation with capsaicin (1 μM), that activates transient receptor potential V1 (TRPV1) and the effect of complex I on the activation of neurons was evaluated by the calcium influx, measured by fluorescence. The percentage of cells responsive to capsaicin was significantly higher in animals pre-stimulated with MSU when compared to saline. Treatment with complex I significantly reduced the percentage of cells responding to capsaicin (Figure 9). Thus, complex I treatment acted directly on cultured DRG neurons reducing their enhanced activity characteristic of gout arthritis.

## Discussion

Gouty arthritis is a debilitating inflammatory condition characterized by intense pain and joint edema. Accumulating MSU crystals in the joint cavity induce the activation of phagocytic cells, such as resident macrophages. There is an increase in pro-inflammatory cytokine production by these cells, attracting neutrophils to the site and, consequently, inducing the production of reactive oxygen species (ROS), activation of NLRP3 and cytokines such as IL-1β, TNF-α, IL-6, IL-18, and chemokines (Busso and So, 2010; Singh, 2016). The main pharmacological therapy for pain in gouty arthritis is the use of non-steroidal anti-inflammatory drugs, biological such as IL-1ra, glucocorticoids and colchicine. However, there may be restriction on the use of these drugs in patients with comorbidities (Engel et al., 2017).

Nitric oxide donor molecules have been used in different pain models, and can trigger analgesic or hyperalgesic effects on neuropathic and post-incisional pain depending on the dose (Sousa and Prado, 2001; Prado et al., 2002; Cury et al., 2011). In the present work, we show that [Ru(bpy)_2_(NO)SO_3_](PF_6_), a NO donor ruthenium complex (complex I) treatment reduced MSU-induced articular pain and edema. To our knowledge, there are no reports in the literature of the use of other NO donors in the treatment of gouty arthritis, thus, this is the first work that demonstrates the beneficial effect of a NO donor complex on gout pain and inflammation. Our study also demonstrated that this effect is due to the NO donor activity of complex, since the empty NO complex I did not reduce pain, edema or inhibit cellular recruitment. It should be noted that at the doses used in this study, the complex I is a safe compound and does not present toxicity, as previously demonstrated by Silva et al. (2010). Thus, this study suggests NO donors as potential pharmacological approaches to reduce gout arthritis.

In fact, previous study demonstrated the analgesic effect of complex I on pain induced by acetic acid, phenyl-p-benzoquinone, complete Freund’s adjuvant, formalin, capsaicin and carrageenan in mice (Staurengo-Ferrari et al., 2013). These effects are dependent, at least in part, on the activation of the cGMP/PKG/ATP-sensitive potassium channel signaling pathway. In this sense, pre-treatment with the inhibitors ODQ, KT5823 or glibenclamide partially reduced the effect of complex I on mechanical hypersensitivity and edema. The activation of the cGMP/PKG/ATP-sensitive potassium channel pathway is critical for peripheral hyperalgesia control, since it leads to hyperpolarization of the nociceptor neurons, decreasing the conduction of the nociceptive stimulus. Stimulating this signaling pathway results in the opening of ATP-sensitive K (+) channels, which is an important mechanism involved in the peripheral effect of analgesic drugs such as dipyrone and morphine (Sachs et al., 2004; Cunha et al., 2010).

The pathology of gout is characterized by intense inflammatory infiltrate in the synovial cavity and adjacent tissues. Indeed, complex I reduced the recruitment of leukocytes (polymorphonuclear and mononuclear cells) observed in the fluid and synovial tissue of animals with gout. These results were corroborated by a decrease of inflammatory cells observed in histological sections and reduced counts of LysM-eGFP^+^ cells in the knee joint synovial fluid. LysM-eGFP^+^ mice expressing cells include mainly neutrophils, but can also encompass macrophages (Faust et al., 2000). Therefore, LysM-eGFP^+^ data demonstrate that complex I reduces the cellular counts of at least two important cells in the pathophysiology of gout.

Neutrophils and macrophages produce ROS in gout. In fact, there is unbalance between endogenous antioxidants, and ROS and reactive nitrogen species (RNS) in gout (Zamudio-Cuevas et al., 2015) and other inflammatory diseases, and pain is related to this unbalance (Failli et al., 2009). There are reports of decreased of superoxide dismutase (SOD) in both animal and patients with osteoarthritis (Regan et al., 2005; Ruiz-Romero et al., 2009). Additionally, the treatment with SOD-analogous compound inhibits pain in CFA or sodium monoiodoacetate-induced arthritis in rats (Di Cesare Mannelli et al., 2013). In cell culture of human fibroblast-like synoviocytes, MSU induces the production of ROS and RNS (Zamudio-Cuevas et al., 2016). In rats, MSU induces neuronal TRPA1 activation by increasing the generation of H_2_O_2_ (Trevisan et al., 2014). Modulation of oxidative stress may be one of the targets of action of NO donors. *In vitro* study with culture of chondrocytes demonstrated that NO can protect these cells against oxidative stress (Del Carlo and Loeser, 2002). In an animal model of diabetic neuropathy, NO donor treatment inhibited oxidative and nitrosative stress and also restored glomerular endothelial NO synthase expression (Hsu et al., 2015). Therefore, we reason it would be rational to evaluated the effect of complex I in MSU-induced oxidative stress. Complex I reduced MSU-induced oxidative stress as observed by increase of FRAP and ABTS activities, normalization of GSH levels and reduction of gp91phox mRNA expression in the knee joint.

Gout inflammation also involves the production of cytokines (Moilanen et al., 2015; So and Martinon, 2017). *In vitro* studies have already shown that MSU induces IL-6 production in cultured human synoviocytes and monocytes (Guerne et al., 1989). The same study showed that synovial fluid from patients with gout exhibits high levels of IL-6. A clinical study has shown that IL-6 levels are increased in individuals with gout, correlating with clinical manifestations of the disease (Cavalcanti et al., 2016). Injection of MSU into the joint of mice increases TNF-α levels, as previously shown (Amaral et al., 2016) and confirmed in this study. Similarly, in mice deficient for TNF-α or its receptors, in addition to decreased pain induced by MSU, inhibition of neutrophil infiltration and production of CXCL1 and IL-1β occurs (Amaral et al., 2016). TNF-α also primes human neutrophils in a manner that these leukocytes start to respond to previously inactive concentrations of MSU as observed by IL-1β secretion (Yokose et al., 2017). Complex I inhibited MSU-induced production of pro-inflammatory cytokines TNF-α and IL-6. TNF-α and IL-6 contribute to the recruitment of inflammatory cells and their activation with the production of ROS and RNS (Lin et al., 2010, 2015; Blaser et al., 2016; Lee et al., 2017). On the other hand, complex I did not alter MSU-induced reduction of IL-10 levels. IL-10 is an anti-inflammatory cytokine and increasing its levels is an approach to reduce inflammation (Li et al., 2013; Ng et al., 2013). Some studies indicate the participation of IL-10 in gout. Treatment of peritoneal macrophages with recombinant human IL-10 decreases the production of CXC chemokine stimulated by MSU crystals (Murakami et al., 2002). In patients with acute gout, there is evidence that increased IL-10 in synovial fluid is related to the spontaneous resolution of the disease (Chen et al., 2011). Nevertheless, complex I did not act via increasing IL-10 levels.

Since the complex I decreases inflammatory cytokines as well as oxidative stress, we evaluate its effect on COX-2 expression. The increase of COX-2 is one of the mechanisms known to be involved in the MSU-induced inflammation. MSU crystals upregulates COX-2 in human monocytes (Pouliot et al., 1998), primary synovial cells and chondrocytes (Lee et al., 2009). Treatment with complex I decreased the MSU-induced cox-2 mRNA expression in the knee joint.

Gout pathophysiology is directly linked to the activation of the so-called signals 1 and 2. Signal 1 refers to the activation of the transcription factor NF-κB, which occurs via ROS and cytokines (Bauernfeind et al., 2009; Busso and So, 2010; Peng et al., 2012). Signal 2 refers to the activation of the NLRP3 inflammasome upon MSU phagocytosis, rupture of phagolysosome and activation of NLRP3 by cathepsin (Martinon et al., 2006; Liu-Bryan, 2010; Rock et al., 2012; Orlowski et al., 2015). NLRP3 inflammasome allows the activation of pro- caspase-1 into active caspase-1 that cleaves pro-IL-1β into IL-1β, which undergoes secretion. IL-1β in turn contributes to inducing inflammation and pain as well as further activation of NF-κB to induce the expression of inflammasome components (NLRP3, ASC, pro-caspase-1, and pro-IL-1β) (Zhang et al., 2016). Complex I inhibited MSU-induced NF-κB activation in the knee joint as observed by the decrease in the total NF-κB p65/phosphorylated NF-κB p65 ratio. This result agrees with the effect of complex I on MSU-induced production of TNF-α and IL-6. Not only in gout arthritis, but in most inflammatory disease, NF-κB regulates the transcription of varied inflammatory molecules (Pahl, 1999; Tak and Firestein, 2001). Inhibiting NF-κB activation is also a mechanism of action of glucocorticoids (Verri et al., 2006). In this sense, drugs that target NF-κB without the side effects of glucocorticoids have a great potential to be useful in varied human inflammatory diseases.

IL-1β is a key cytokine in gout inflammation considering the importance of NLRP3 (Mitroulis et al., 2013). We observed that MSU-induced an increase of pro-IL-1β expression *in vivo*, which was diminished by complex I. This result agrees with the complex I inhibition of MSU-induced NF-κB activation. Inhibition of the NF-κB signaling pathway appears to be at least one of the mechanisms involved in modulating NO on inflammation, since treatment with NO donors decreases the expression of adhesion molecules by inhibiting activation of NF-κB in human saphenous vein (De Caterina et al., 1995) and human umbilical vein endothelial cells (Waldow et al., 2006).

Complex I also inhibited MSU-induced IL-1β production and pro-il-1β and nlrp3 mRNA expression in the knee joint. Further, we used an *in vitro* assay designed to evaluate MSU-induced NLRP3 inflammasome activation (Martinon et al., 2006; Zheng et al., 2015). Bone marrow derived macrophages (BMDM) received as signal 1 lipopolysaccharide of Gram-negative bacteria (LPS). BMDM also received signal 2 (MSU), which induced the release of IL-1β in the culture media. BMDM were treated with complex I after LPS stimulus/before MSU stimulus to determine if the NO donor could inhibit inflammasome activation at a time point in which signal 1 has already been effective. Complex I significantly inhibited MSU-induced release of IL-1β in the culture media indicating that the NO donor effect involves inhibition of inflammasome activation. This is an important mechanism considering the effectiveness of anti-IL-1 therapies for the treatment of human gout arthritis (So et al., 2007).

The importance of TRPV1 activation has been demonstrated in MSU-induced acute gout attacks (Ramonda et al., 2014). The immunoreactivity of the TRPV1 receptor in the joint tissue of animals stimulated with MSU crystals is increased (Hoffmeister et al., 2014). Treatment with selective TRPV1 antagonists as well as desensitization or defunctionalization of afferent fibers sensitive to capsaicin inhibits MSU-induced pain and edema in rats (Hoffmeister et al., 2011, 2014). Plasma extravasation, leukocyte infiltration and articular fluid IL-1β production induced by MSU are also decreased by the TRPV1 antagonist SB366791 (Hoffmeister et al., 2014). Therefore, considering the importance of TRPV1 activation in the pathophysiology of gout arthritis, we evaluated the effect of complex I in capsaicin-induced calcium influx in DRG neurons of mice that were stimulated with MSU. Complex I inhibited the TRPV1 activation-induced calcium influx of capsaicin responsive neurons harvested from mice with gout arthritis.

*In vitro* studies comparing NO release capacity demonstrated that Complex I is capable of releasing NO after reacting with thiols and presents superior physico-chemical properties and stability compared to other metal complexes (Silva et al., 2006, 2011). In addition to NO, the thiol reaction with metallonitrosyl complexes, such as Complex I, can generate HNO (nitroxyl) (Silva Sousa et al., 2016). Nitroxyl donors have also analgesic, anti-inflammatory and microbicide actions (Zarpelon et al., 2013; Staurengo-Ferrari et al., 2014b, 2017; Longhi-Balbinot et al., 2016). The release of NO by Complex I is mediated by a reaction involving thiols such as cysteine and glutathione (Silva et al., 2011). Since the development of novel NO donor metallic complexes has been the subject of recent studies, the literature on the pharmacokinetics of the Complex I, for example, has not yet been reported. Therefore, ruthenium NO complexes represent a topic that deserves further investigation.

## Conclusion

To our knowledge, this is the first demonstration that treatment with a ruthenium complex NO donor inhibits MSU-induced inflammation and pain, thus, suggesting this NO donation using ruthenium complex as a promising therapeutic approach in the management of gout inflammation. The beneficial effect of complex I {[Ru(bpy)_2_(NO)SO_3_](PF_6_)} in gout depends on the activation of the cGMP/PKG/ATP-sensitive potassium channel signaling pathway and inhibition of NF-κB activation. The mechanism of action of complex I also depends on the inhibition of MSU-induced inflammasome activation and reducing neuronal activation. Therefore, the present data suggest a promising therapeutic property of complex I, given it targets the main pathophysiological mechanisms of gout arthritis. The multitarget mechanism of action with direct analgesic actions (present data) and safe profile at the present dose (Silva et al., 2010) support that complex I is a good candidate for further pre-clinical and clinical studies toward clinical trials.

## Author Contributions

AR, DL-B, MB, VF, CS-V, SB-G, TZ, LS-F, SB, TC, AB and FG contributed to the data collection and analysis. AR, LL, RC, and WV contributed to the literature database search, data analysis, and writing of the manuscript. WV supervised the study. All authors read and approved the final version of the manuscript.

## Conflict of Interest Statement

The authors declare that the research was conducted in the absence of any commercial or financial relationships that could be construed as a potential conflict of interest.
