# Supplementary material for: [Ru(bpy)2(NO)SO3](PF6), a Nitric Oxide Donating Ruthenium Complex, Reduces Gout Arthritis in Mice
Source: Front Pharmacol. 2019 Mar 12;10:229. doi: 10.3389/fphar.2019.00229 (PMC6423075; doi:10.3389/fphar.2019.00229)
Supplement: Supplementary file 1 [file Presentation_1.PDF]

Supplementary Material

$[Ru(bpy)_2(NO)SO_3](PF_6)$, a nitric oxide donating ruthenium complex, reduces gout arthritis in mice.

Ana C. Rossaneis[1], Daniela T. Longhi-Balbinot[1], Mariana M. Bertozzi[1], Victor Fattori[1], Carina Z. Segato-Vendrameto[1], Stephanie Badaro-Garcia[1], Tiago H. Zaninelli[1], Larissa Staurengo-Ferrari[1], Sergio M. Borghi[1], Thacyana T. Carvalho[1], Allan J. C. Bussmann[1], Florêncio S. Gouveia Júnior[2], Luiz G. F. Lopes[2], Rubia Casagrande[3], Waldiceu A. Verri, Jr[1*]

[1]Laboratory of Pain, Inflammation, Neuropathy, and Cancer, Department of Pathology, Londrina State University, Londrina, PR, Brazil

[2]Department of Organic and Inorganic Chemistry, Federal University of Ceará, Fortaleza, CE, Brazil.

[3]Department of Pharmaceutical Sciences, University Hospital (Health Science Centre), Londrina State University, PR, Brazil.

*** Correspondence:**
Waldiceu A. Verri Jr, Ph.D.
waverri@uel.br or waldiceujr@yahoo.com.br

SUPPLEMENTARY DATA

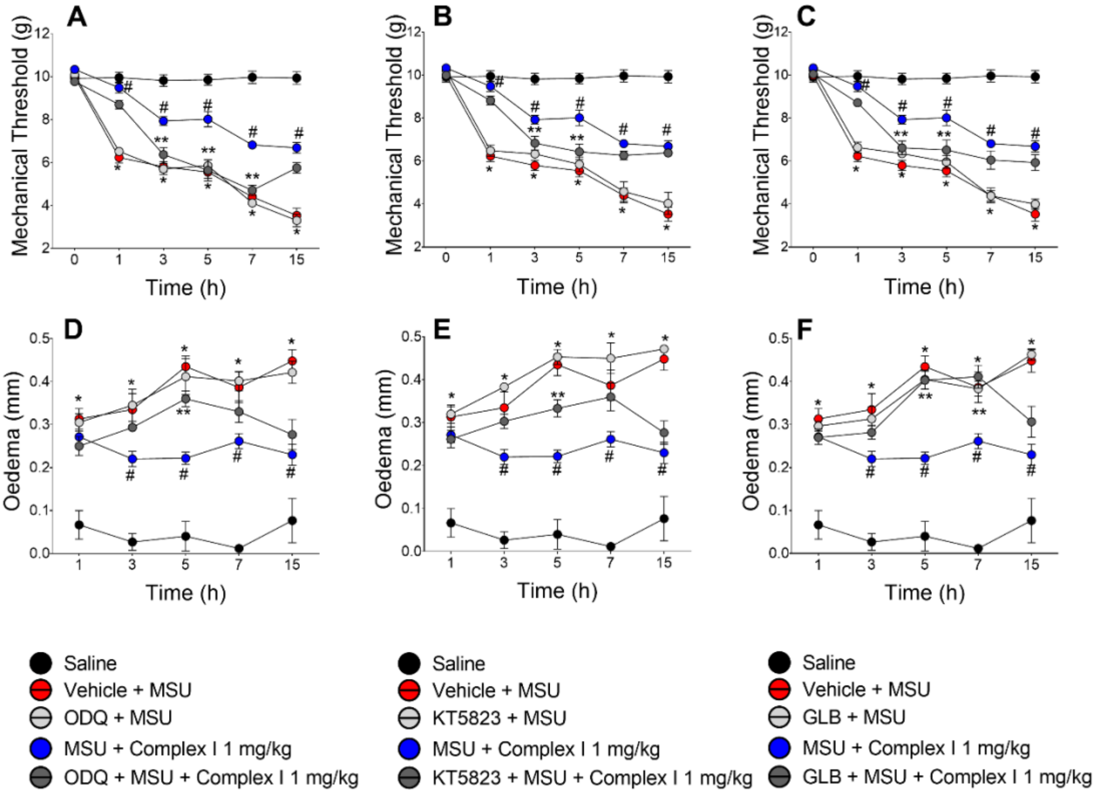

Figure S1. Complex I reduces MSU-induced mechanical hypersensitivity and edema by activating the cGMP/PKG/ATP-sensitive potassium channel pathway. Pharmacological treatments targeting guanylate cyclase (ODQ; 0.3 mg/kg, i.p., 30 min before complex I treatment, **A** and **D**), PKG (KT5923; 0.5µg/mouse, i.p., 5 min before, **B** and **E**) and ATP-sensitive potassium channel (glibenclamide - GLB; 0.3 mg/kg, i.p., 45 min before, **C** and **F**) were administrated before complex I treatment, and mechanical hypersensitivity **(A, B and C)** and edema **(D, E and F)** were evaluated 1, 3, 5, 7, and 15 h after MSU stimulus. Results are expressed as mean ± SEM (n = 6 per group per experiment, representative of two experiments). *p < 0.05 compared to saline group, #p < 0.05 compared to vehicle + MSU group **p < 0.05 compared to MSU + complex I. Two-way ANOVA followed by Tukey's test.
